# The association of insulin resistance and obesity with uterine fibroids in non-diabetic populations: a cross-sectional study

**DOI:** 10.1038/s41598-025-30265-1

**Published:** 2025-11-25

**Authors:** Danting Sun, Haixia Liu, Jiaze Gao, Yapei Su, Yuance Xu, Teng Lv

**Affiliations:** 1 Department of Gynecology, The Affiliated Hospital of Qingdao University, Qingdao, Shandong China; 2 The Affiliated Qingdao Third People’s Hospital of Qingdao University, Qingdao, Shandong China

**Keywords:** Insulin resistance, Uterine fibroids, HOMA-IR, NHANES, Physiology, Diseases, Endocrinology, Medical research, Risk factors

## Abstract

To investigate the association between insulin resistance (IR) and obesity and uterine fibroids (UFs) in non-diabetic populations using data from the National Health and Nutrition Examination Survey (NHANES). This cross-sectional study analyzed data from NHANES (1999–2006) and included 867 non-diabetic women aged 20–54 years. IR was assessed using the triglyceride-glucose (TyG) index, TyG-waist-to-height ratio (TyG-WHtR), TyG-body mass index (TyG-BMI), and the homeostasis model assessment of insulin resistance (HOMA-IR). Obesity was assessed by WHtR, weight-adjusted waist index (WWI), waist circumference (WC) and BMI. Logistic regression, restricted cubic spline curves (RCS), subgroup analyses, and interaction tests were performed to evaluate the associations. HOMA-IR index (OR = 1.17, *p* = 0.004) and BMI (OR = 1.04, *p* = 0.036) were significantly positively correlated with UFs. Subgroup analyses in those not using female hormones show that among women aged 20–38 years, the association between BMI and UFs was stronger (*P* < 0.05), while in women aged 39–54 years, HOMA-IR was stronger (*P* < 0.05). The RCS models indicated significant positive linear associations between HOMA-IR, as well as BMI, and UFs. Our study demonstrates that IR and BMI are independently and positively associated with the presence of UFs in non-diabetic women, indicating that they may be potentially modifiable factors associated with UFs development.

## Introduction

Uterine fibroids (UFs), benign tumors arising from the smooth muscle cells of the uterus, represent the most prevalent gynecological neoplasms in female of reproductive age^1^. Although their exact etiology remains incompletely understood, emerging evidence implicates a multifactorial pathogenesis involving genetic predisposition, sex hormone dysregulation, and growth factor signaling^2,3^. Estrogen and progesterone drive UFs proliferation via receptor-mediated pathways, while different growth factors perform actions in myometrium and in leiomyomas^4^.

Metabolic factors may play an important role in the occurrence of UFs. Results of meta-analysis suggest that obesity may increase the risk/prevalence of UFs, and the association is non-linear^5^. Adipose tissue can contribute to higher estrogen levels, thereby promoting fibroid growth^6^.

Insulin resistance (IR) manifests through diminished insulin efficacy in facilitating glucose absorption and utilization within peripheral tissues, resulting in a compensatory increase in insulin levels^7^. This metabolic dysfunction has been implicated in various pathological processes, including inflammation, endothelial dysfunction, and hormonal imbalances, all of which may contribute to the development and growth of UFs^8^. Notably, recent investigations have highlighted IR—a hallmark of metabolic dysfunction—as a potential modulator of UFs pathogenesis^9^.

Experimental models provide compelling biological plausibility for this association. In estrogen- and progesterone-treated female rats with diet-induced IR, uterine myometrial hyperplasia was markedly exacerbated, accompanied by upregulated expression of estrogen receptor (ER), progesterone receptor (PR), and proliferative markers such as proliferating cell nuclear antigen (PCNA)^10,11^. These findings suggest that IR may amplify sex hormone-driven mitogenic signaling in uterine tissue. Furthermore, IR-associated hyperinsulinemia potentiates the activity of insulin-like growth factor-I (IGF-I), a mitogen implicated in UFs cell proliferation^12,13^.

Clinical observations further suggest an interplay between IR and UFs progression^14^. Serum levels of IGF-I and inhibition of vascular endothelial growth factor (VEGF)—key mediators of UFs growth—exhibit dynamic changes following uterine artery embolization, with IR status correlating with treatment response and fibroid volume reduction^15,16^. Moreover, IR prevalence escalates in women with endometrial pathologies^17^, implying systemic metabolic disturbances may concurrently influence uterine neoplasia^18^. Nevertheless, current evidence remains fragmented, limited by heterogeneous diagnostic criteria for IR and insufficient adjustment for confounders like adiposity and inflammation^19^. Human epidemiological data also reveal contradictory trends^20^. A cross-sectional analysis of women aged 35–49 years demonstrated an inverse relationship between hyperinsulinemia and UFs risk among Black participants, with no significant association observed in white populations^21^. This racial disparity underscores the complexity of IR-UF interactions, potentially mediated by vascular dysfunction or unmeasured confounders.

The contradictions in existing research require strict epidemiological research to clarify^22^. This cross-sectional research intends to explain the association of IR and obesity with UFs in a large, diverse cohort, employing standardized homeostasis model assessment of insulin resistance (HOMA-IR) thresholds and comprehensive adjustment for metabolic covariates. By resolving these inconsistencies, our findings may inform targeted prevention strategies for UFs susceptibility populations.

## Materials and methods

### Cross-sectional survey

#### Study design and participants

**Fig. 1:**
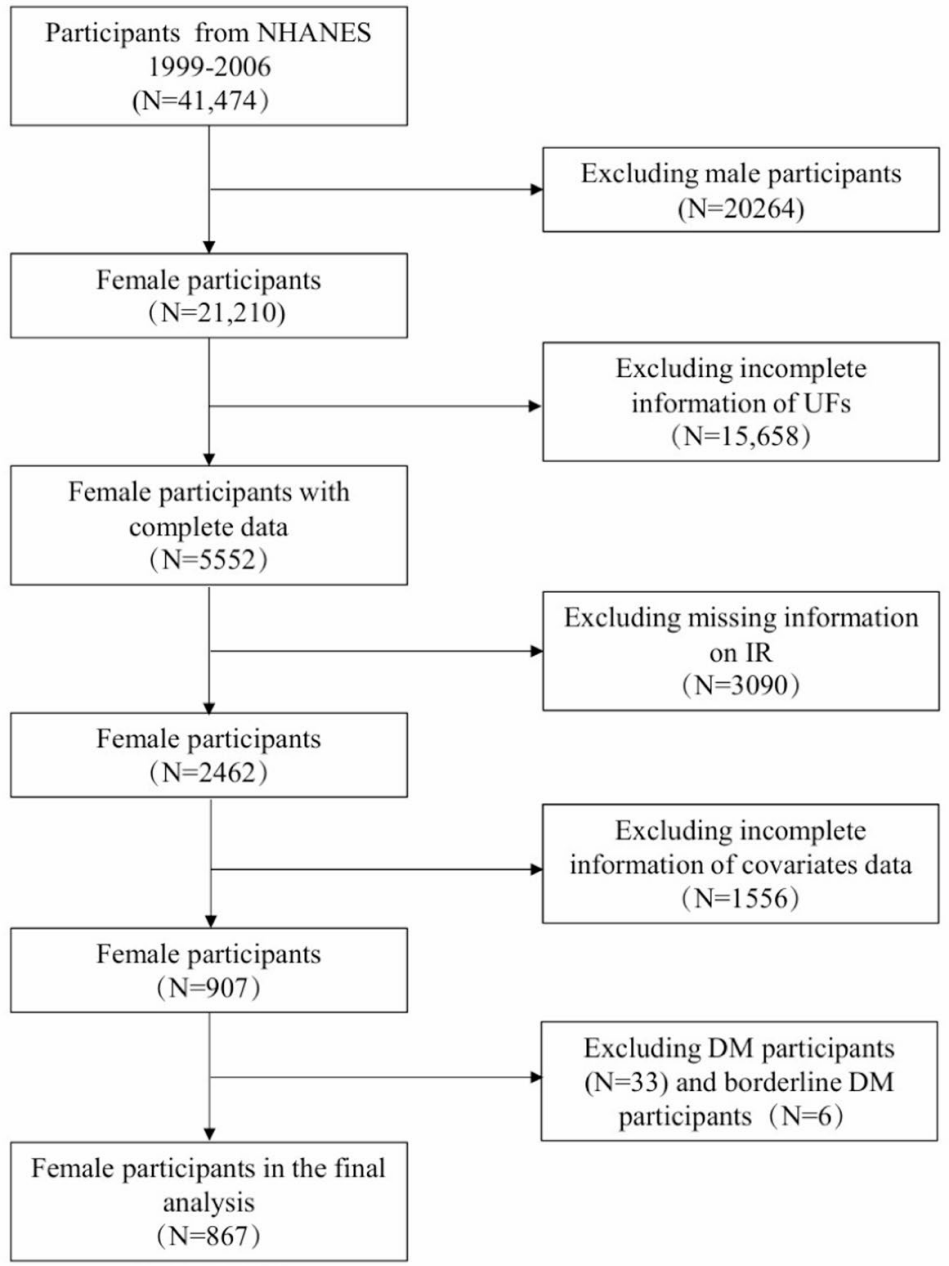
Flowchart of participant selection.

This study was executed following the guidelines set forth by the Strengthening the Reporting of Observational Studies in Epidemiology (STROBE)^23^. A completed STROBE checklist is provided as Supplementary Material. The NHANES databases, which include data from 1999 to 2006, were examined. The NCHS Research Ethics Review Board approved this significant study, which included 21,210 women in the surveys. Written informed consent was obtained from all participants. Given that this secondary analysis utilized anonymized data, it was deemed exempt from further ethical review. We excluded 20,340 participants with incomplete data and an additional 39 individuals with diabetes or pre-diabetes, resulting in a final analytic sample of 867 non-diabetic individuals aged 20–54 years with complete clinical, anthropometric, and laboratory information. We compared the baseline characteristics of the excluded and included populations and found systematic differences in age. Since most of the data we excluded were from non-UFs high-incidence individuals under 20 years old, we believe these data have a relatively minor impact on the results. The detailed flow diagram is illustrated in Fig. 1. All methods were performed in accordance with the relevant guidelines and regulations.

#### Data collection

Age, race, education, marriage, poverty income ratio (PIR), female hormone use, number of pregnancies, body mass index (BMI), waist circumference (WC), height, weight, hip circumference, triglycerides (TG), serum creatinine, fasting plasma glucose (FPG), and fasting insulin.

(FINS) were collected. Waist-to-height ratio (WHtR), weight-adjusted waist index (WWI), and estimated glomerular filtration rate (eGFR) was calculated. The assessment of alcohol use, diabetes, smoking status, diabetes and hypertension was based on self-reported diagnoses. Low physical activity was delineated as < 500 MET-minutes per week^24^.The dietary inflammatory index, a measure of the inflammatory potential of diet, was assessed in this study based on 27 food components^25^.

#### IR-related indices

To assess IR, we measured several indices related to IR, which were defined as follows^26–28^:

$${\mathrm{TyG}}\,{\mathrm{index}}\, = \,{\text{Ln }}\left( {{\text{fasting TG }}\left[ {{\mathrm{mg}}/{\mathrm{dL}}} \right]\, \times \,{\text{FPG }}\left[ {{\mathrm{mg}}/{\mathrm{dL}}} \right]/{\mathrm{2}}} \right)$$

$${\mathrm{TyG}} - {\mathrm{BMI}}\, = \,{\mathrm{TyG}}\, \times \,{\mathrm{BMI}}$$

$${\mathrm{TyG}} - {\mathrm{WHtR}}\, = \,{\mathrm{TyG}}\, \times \,{\mathrm{WHtR}}$$

$${\mathrm{HOMA}} - {\mathrm{IR}}\, = \,{\mathrm{FINS}}\left( {\upmu {\mathrm{U}}/{\mathrm{mL}}} \right){\text{ x FPG }}\left( {{\mathrm{mmol}}/{\mathrm{L}}} \right){\text{ }}/{\text{ 22}}.{\mathrm{5}}$$

#### Statistical analysis

We evaluated the normality of the data with the Shapiro-Wilk test, which indicated a non-normal distribution. To compare the groups, we utilized the Wilcoxon rank sum test, presenting the results as medians and interquartile ranges. Categorical data were analyzed with chi-squared tests, presenting frequencies and proportions^29^. Univariate and multivariate regression analyses were conducted to evaluate the associations between covariates and UFs. Four logistic regression models were created. Four logistic regression models were created. Model 1: Unadjusted; Model 2: Adjust for ethnicity, marriage, education, age, PIR; Model 3: Model 2 + lifestyle factors and health indicators (smoking status, alcohol, physical activities, dietary inflammatory index, hypertension, eGFR); Model 4: Model 3 + reproductive health (female hormone use, number of pregnancies). To assess the associations, restricted cubic spline curves (RCS), subgroup analyses, and interaction tests were employed. All analyses were conducted using R software 4.3.1 (available at https://www.R-project.org) with the “survey” and “rms” packages, and the Zstats 1.0 online platform for additional outputs. *P* < 0.05 is considered significant.

## Results

### Baseline characteristics of the participants

The research involved 867 non-diabetic women aged 20–54, with 738 having no UFs and 129 having UFs. Baseline features of the participants were shown in Table 1. The UFs group was older (median age 45 vs. 37 years, *p* < 0.001) and had a higher median PIR (4.72 vs. 3.39, *p* < 0.001) and lower eGFR (103.35 vs. 105.45, *p* < 0.001). This economic advantage likely enhances healthcare access and health awareness, leading to more frequent screenings and consequently higher UFs detection rates^30^.The UFs group also had a higher prevalence of female hormone use (34.55% vs. 12.02%, *p* < 0.001) and fewer participants with less than high school education (5.49% vs. 11.50%, *p* = 0.048). No significant differences were observed in BMI, WC, WHtR, parity, smoking status, alcohol use, physical activity, or the dietary inflammatory index .

**Table 1:** Baseline features of the participants.

Variable	Without UFs (*n* = 738)	UFs (*n* = 129)	*P*
Age	37.00 (31.00, 46.00)	45.00 (41.00, 50.00)	< 0.001***
BMI	26.23 (22.95, 31.00)	27.18 (23.17, 31.92)	0.187
WC	88.60 (80.20, 98.70)	91.00 (81.60, 102.10)	0.504
WWI	10.65 (10.15, 11.19)	10.56 (10.05, 11.13)	0.582
WHtR	0.55 (0.49, 0.61)	0.55 (0.49, 0.63)	0.502
eGFR	105.45 (91.97, 116.88)	103.35 (91.40, 109.30)	< 0.001***
TyG	8.38 (8.06, 8.78)	8.46 (8.11, 8.84)	0.230
TyG BMI	222.32 (187.33, 265.23)	235.63 (198.73, 282.53)	0.167
TyG WHtR	4.62 (4.01, 5.27)	4.80 (4.08, 5.44)	0.349
HOMA IR	1.73 (1.14, 2.52)	2.05 (1.13, 3.26)	0.125
Female hormone use	67 (12.02%)	41 (34.55%)	< 0.001***
Number of pregnancies	3.00 (2.00, 4.00)	3.00 (2.00, 4.00)	0.614
PIR	3.39 (1.82, 5.00)	4.72 (2.89, 5.00)	< 0.001***
Hispanic ethnicity	159 (9.42%)	19 (6.73%)	0.232
Married or living with partner	553 (76.28%)	95 (81.11%)	0.358
Smoking status	298 (43.79%)	55 (51.84%)	0.248
Alcohol use	490 (72.21%)	86 (74.63%)	0.569
Hypertension	120 (19.40%)	34 (20.44%)	0.797
Less than high school	123 (11.50%)	9 (5.49%)	0.048*
Low physical activity	317 (40.81%)	49 (37.00%)	0.473
Dietary inflammatory index	1.63 (0.34, 2.83)	1.57 (0.35, 2.56)	0.217

Hispanic Ethnicity: Mexican American and Other Hispanic.

*p < 0.05, **p < 0.01, ***p < 0.001.

### Association between covariates and UFs

**Fig. 2:**
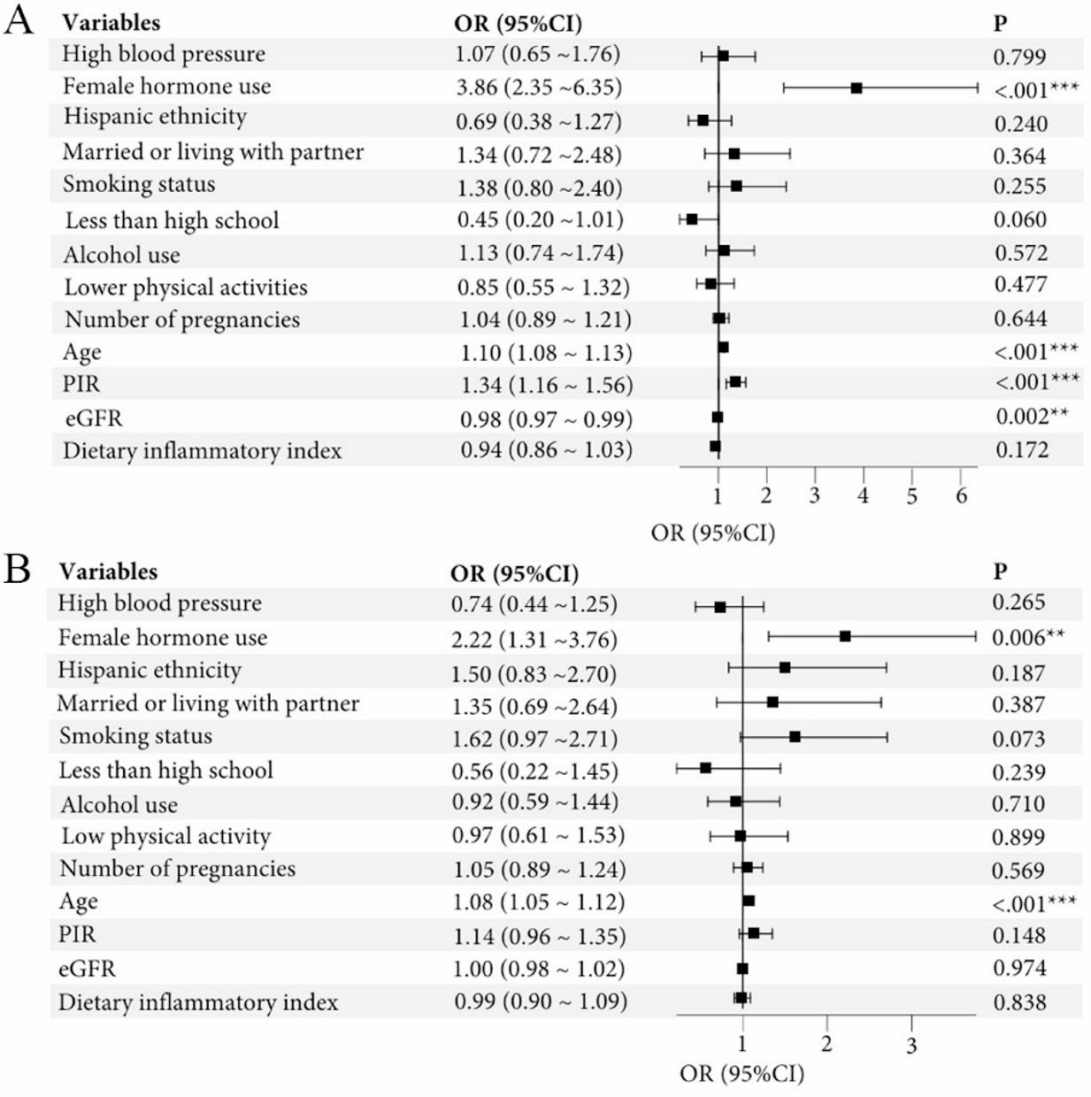
The association between covariates and UFs in univariate and multivariate regression analysis. *p < 0.05, **p < 0.01, ***p < 0.001.

The association of covariates with UFs in the study is shown in Fig. 2. In univariate regression analysis, female hormone use (OR = 3.86, 95% CI: 2.35 ~ 6.35, *p* < 0.001), age (OR = 1.10, 95% CI: 1.08 ~ 1.13, *p* < 0.001), PIR (OR = 1.34, 95% CI: 1.16 ~ 1.56, *p* < 0.001), and eGFR (OR = 0.98, 95% CI: 0.97 ~ 0.99, *p* = 0.002) were all significantly associated with UFs. In addition, in the multivariate regression analysis, female hormone use (OR = 2.22, 95% CI: 1.31 ~ 3.76, *p* = 0.006) and age (OR = 1.08, 95% CI: 1.05 ~ 1.12, *p* < 0.001) remained significantly correlated with UFs, while PIR and eGFR were not significant. Other variables such as high blood pressure, ethnicity, marital status, education, alcohol use, physical activity, and dietary inflammatory index did not show significant associations with UFs in either analysis.

### Associations between the indicators of IR and UFs

The relationship between IR indicators and UFs was examined using multiple logistic regression models. As shown in Table 2, the HOMA-IR index exhibited a significant and progressive association with UFs across all models, with the OR increasing from 1.09 (95% CI: 0.99 ~ 1.20, *p* = 0.081) in Model 1 to 1.17 (95% CI: 1.06 ~ 1.30, *p* = 0.004) in Model 4. BMI was significantly associated with UFs starting from Model 3 (OR = 1.04, 95% CI: 1.01 ~ 1.08, *p* = 0.047) and remained significant in Model 4 (OR = 1.04, 95% CI: 1.01 ~ 1.08, *p* = 0.036). Other indicators such as WWI, WHtR, TyG, TyG-BMI and TyG-WHtR did not demonstrate significant associations with UFs in any model. These findings suggest that HOMA-IR and BMI are potentially important predictors for UFs risk in non-diabetic women.

**Table 2:** Multivariate logistic regression analysis of the relationship between IR and UFs.

Variables	Model1		Model2		Model3		Model4	
	OR (95%CI)	*P*	OR (95%CI)	*P*	OR (95%CI)	*P*	OR (95%CI)	*P*
WWI	0.91 (0.66 ~ 1.26)	0.569	0.86 (0.60 ~ 1.22)	0.392	0.83 (0.57 ~ 1.22)	0.356	0.81 (0.57 ~ 1.17)	0.278
WHtR	2.58 (0.18 ~ 36.12)	0.486	2.00 (0.09 ~ 42.62)	0.660	3.37 (0.15 ~ 74.44)	0.446	3.75 (0.16 ~ 85.72)	0.414
BMI	1.03 (0.99 ~ 1.06)	0.123	1.03 (0.99 ~ 1.07)	0.212	1.04 (1.01 ~ 1.08)	0.047*	1.04 (1.01 ~ 1.08)	0.036*
TyG	1.19 (0.82 ~ 1.73)	0.360	1.06 (0.71 ~ 1.56)	0.785	1.03 (0.66 ~ 1.59)	0.910	1.00 (0.64 ~ 1.56)	0.998
TyG- BMI	1.00 (1.00 ~ 1.01)	0.118	1.00 (1.00 ~ 1.01)	0.241	1.00 (1.00 ~ 1.01)	0.070	1.00 (1.00 ~ 1.01)	0.059
TyG- WHtR	1.11 (0.87 ~ 1.42)	0.413	1.06 (0.80 ~ 1.41)	0.671	1.10 (0.82 ~ 1.48)	0.508	1.11 (0.83 ~ 1.49)	0.498
HOMA IR	1.09 (0.99 ~ 1.20)	0.081	1.12 (1.03 ~ 1.23)	0.017*	1.16 (1.05 ~ 1.28)	0.007**	1.17 (1.06 ~ 1.30)	0.004**

Model1: Crude.

Model2: Adjust for ethnicity, marriage, education, age, PIR.

Model3: Adjust for high blood pressure, ethnicity, marriage, smoking status, education, alcohol use, low physical activities, age, PIR, eGFR, dietary inflammatory index.

Model4: Adjust for high blood pressure, female hormone use, ethnicity, marriage, smoking status, education, alcohol use, low physical activities, number of pregnancies, age, PIR, eGFR, dietary inflammatory index.

OR: Odds Ratio, CI: Confidence Interval.

*p < 0.05, **p < 0.01, ***p < 0.001.

### Subgroup analysis and interaction effects

**Fig. 3:**
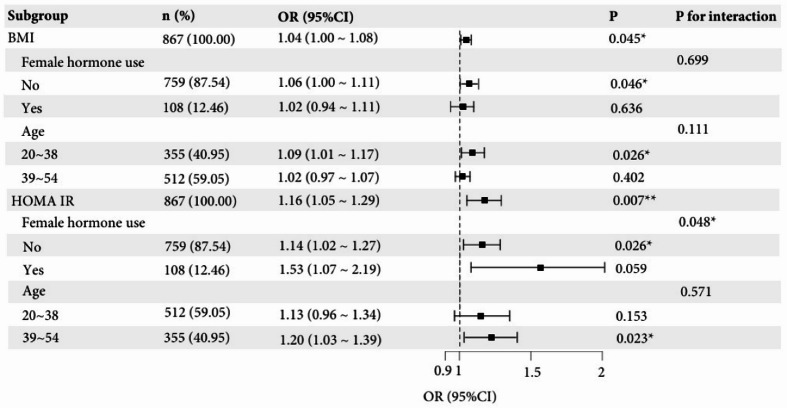
Subgroup analysis of the relationship between BMI, HOMA-IR with UFs. *p < 0.05, **p < 0.01, ***p < 0.001.

Subgroup analyses were performed based on age and female hormone use, as these were found to be significantly associated with UFs in the multivariate regression analysis. The classification of age was based on the median age of the analyzed population. Subgroup analyses were exploratory and uncorrected for multiple testing. Figure 3 presents the outcomes of these subgroup analyses of the relationship between BMI/HOMA-IR and UFs.

Among women aged 20–38 years, the association between BMI and UFs was stronger in those not using female hormones (*P* < 0.05). The association between BMI and UFs was not influenced by age or female hormone use (interaction *P* > 0.05). In women aged 39–54 years, the association between HOMA-IR and UFs was stronger in those not using female hormones (*P* < 0.05). The relationship between HOMA-IR and UFs was not affected by age (interaction *P* > 0.05), but it was influenced by female hormone use (interaction *P* = 0.048).

#### RCS analysis

**Fig. 4:**
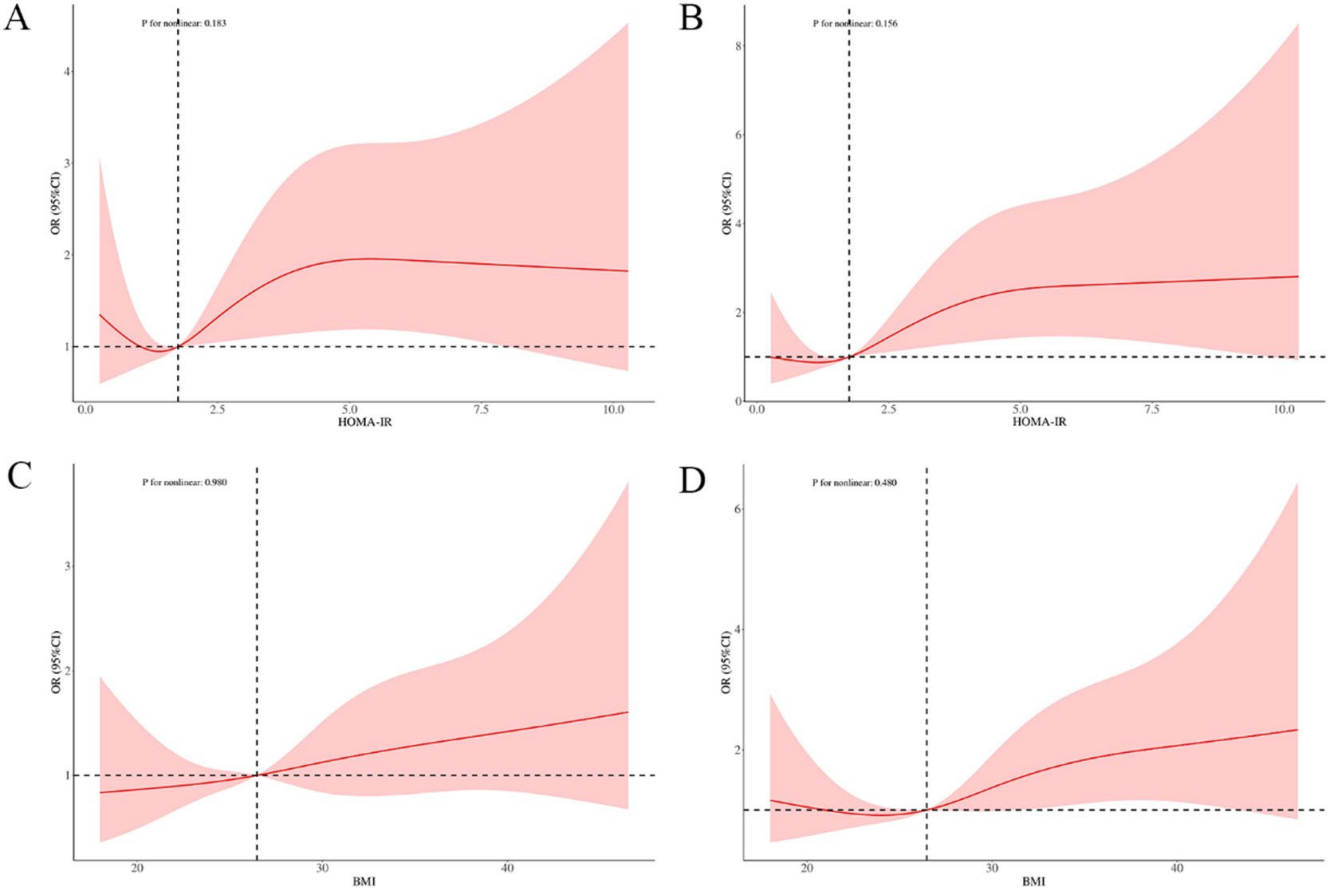
Analysis of RCS model associated with HOMA-IR and UF. (**A**) RCS analysis of the association between HOMA-IR and UFs in the unadjusted model. (**B**) RCS analysis of the association between HOMA-IR and UFs in the fully adjusted model. (**C**) RCS analysis of the association between BMI and UFs in the unadjusted model. (**D**) RCS analysis of the association between BMI and UFs in the fully adjusted model.

The RCS models indicated significant positive linear associations between HOMA-IR and UFs (Unadjusted: P for nonlinearity = 0.183, Fig. 4A; Fully Adjusted: P for nonlinearity = 0.156, Fig. 4B). Consistent evidence for linearity was also observed for BMI and UFs in both the unadjusted (P for nonlinearity = 0.980, Fig. 4C) and fully adjusted models (P for nonlinearity = 0.480, Fig. 4D). These significant positive linear associations suggest that both HOMA-IR and BMI exert a continuous, dose-dependent effect on UF risk. The consistency across both unadjusted and fully adjusted models underscores the robustness of these linear associations.

## Discussion

Our study provides evidence that both BMI and HOMA-IR are significantly correlated with the presence of UFs in non-diabetic women. The current findings highlight the importance of considering metabolic health, particularly IR, in the context of UFs, potentially guiding future preventive and therapeutic strategies.

The positive correlation between HOMA-IR and UFs suggests that IR may be a significant factor for the development of UFs. This finding aligns with previous research, such as a cohort study involving 2,570 participants from the Study of Women’s Health Across the Nation (SWAN), which reported that higher levels of insulin and IR were associated with an increased incidence of fibroid diagnoses, particularly in premenopausal women^31^. Similarly, a study of 2,249 patients indicated that women with polycystic ovary syndrome (PCOS), characterized by IR, exhibited a lower prevalence of non-cavity-distorting fibroids compared to those with unexplained infertility, highlighting the complex interplay between metabolic conditions and fibroid development^32,33^. In contrast, some studies have yielded inconsistent results. For instance, a study examining 1,230 participants revealed no significant correlation between IR and the presence of UFs, indicating that other factors might be involved in the development of fibroids^34^. The discrepancies might stem from variations in research methodology, sample dimensions, and the characteristics of the study cohorts, thereby highlighting the necessity for additional research into the complex, multifactorial etiology of UFs^35,36^.

The biological mechanisms by which IR could influence UFs growth are multifaceted. IR is correlated with chronic low-grade inflammation, which can lead to increased production of cytokines and growth factors that stimulate fibroid cell proliferation^37,38^. Additionally, IR can disrupt normal insulin signaling pathways, leading to an imbalance in the production of hormones such as estrogen and IGF-I, which are known to promote fibroid growth^39^. Furthermore, IR may enhance the activity of growth factors like VEGF, contributing to angiogenesis within fibroids and thus supporting their growth and development^40^. Studies indicate that IR significantly influences the formation of UFs through its effects on hormonal levels and cellular proliferation mechanisms. Specifically, the association between elevated HOMA-IR scores and the prevalence of UFs suggests that IR may promote the proliferation of specific cell types involved in fibroid development, such as smooth muscle cells and fibroblasts^41,42^. This aligns with previous research that has identified a similar cellular subtype characterized by heightened sensitivity to insulin signaling, which may contribute to the pathogenesis of UFs^43,44^. Future investigations should focus on elucidating the key transcription factors that regulate this specific cell subtype, as understanding their roles could provide insights into the molecular pathways driving fibroid growth^45^. Identifying these transcription factors and their functional implications will be crucial for developing targeted therapeutic strategies aimed at mitigating the effects of IR on uterine fibroid formation and progression^46,47^. Further research is warranted to explore the interplay between insulin signaling, cellular proliferation, and the hormonal environment in the context of UFs, which may ultimately lead to novel interventions for affected patients.

Future research should focus on exploring the interactions between UFs and insulin signaling, cell proliferation, and hormonal environment, which may ultimately lead to new interventions for affected patients.

Our research has found that BMI, a widely recognized marker of obesity, is a risk factor for UFs, which aligns with previous research suggesting that excess adipose tissue can contribute to higher estrogen levels, thereby promoting fibroid growth^6^. Subgroup analyses show that among women aged 20–38 years, the association between BMI and UFs was stronger. While in women aged 39–54 years, the association between HOMA-IR and UFs was stronger. This provides new ideas for the prevention and management of UFs throughout their entire lifecycle.

This study presents significant findings regarding the relationship between IR indicators, specifically the HOMA-IR and BMI, and the occurrence of UFs. NHANES is a mature and widely used epidemiological resource with strict data collection and quality assurance protocols. Previous studies have verified its reliability in assessing the association between exposure and health outcomes, providing confidence in the validity of the results of this study^48,49^. Nevertheless, it is crucial to recognize certain intrinsic constraints of the present research. The most significant limitation of this study is the possibility of considerable selection bias. Our analysis sample only included a portion of the initial NHANES queue, mainly due to extensive exclusion of participants because of missing data. The drastic reduction in sample size may result in participants not being representative of a wider population. Therefore, the observed association between HOMA-IR, BMI, and UF may not be generalizable to other populations without further validation. Secondly, the study draws on NHANES data and relies on self-reported diagnoses of UFs, a factor that could introduce reporting bias. Subgroup analyses were exploratory and hypothesis-generating due to small sample sizes and lack of multiple testing correction. Additionally, the sample size may not be sufficiently large to generalize the findings across diverse populations, and potential batch effects in the datasets could introduce variability that affects the stability of the findings. While our study provides valuable insights into the relationship between IR and UFs, it is important to acknowledge that our findings are based on cross-sectional data, and no causal inference can be established regarding the relationship between IR, BMI, and UF. Subsequent investigations should encompass more extensive longitudinal studies to verify these associations and elucidate the underlying mechanisms.

In conclusion, our study cross-sectional study suggests an association between HOMA-IR, BMI and UFs in non-diabetic women. The associations of BMI and HOMA-IR on UFs risk appears to vary across age groups, suggesting a potential need for age-specific strategies in UFs prevention and management. Future longitudinal investigations are essential to elucidating the causal relationships and the underlying mechanisms linking IR and UFs, with consideration given to the differential effects of BMI and HOMA-IR across the lifespan.

## Data Availability

The data utilized in this research were sourced from the NHANES database (https://wwwn.cdc.gov/nchs/nhanes/Default.aspx). Requests for access to the data used in this study should be directed to the corresponding author.
